# Intervention To Stop Transmission of Imported Pneumonic Plague — Uganda, 2019

**DOI:** 10.15585/mmwr.mm6909a5

**Published:** 2020-03-06

**Authors:** Titus Apangu, Sarah Acayo, Linda A. Atiku, Harriet Apio, Gordian Candini, Felix Okoth, John Kaggwa Basabose, Lawrence Ojosia, Sam Ajoga, Grace Mongiba, Milton Makoba Wetaka, Joshua Kayiwa, Stephen Balinandi, Amy Schwartz, Brook Yockey, Christopher Sexton, Elizabeth A. Dietrich, Ryan Pappert, Jeannine M. Petersen, Paul S. Mead, Julius J. Lutwama, Kiersten J. Kugeler

**Affiliations:** ^1^Uganda Virus Research Institute, Arua and Entebbe, Uganda; ^2^Uganda Ministry of Health, Zombo District, Uganda; ^3^Uganda Public Health Emergency Operations Center, Kampala, Uganda; ^4^Division of Vector-Borne Diseases, National Center for Emerging and Zoonotic Infectious Diseases, CDC.

Plague, an acute zoonosis caused by *Yersinia pestis,* is endemic in the West Nile region of northwestern Uganda and neighboring northeastern Democratic Republic of the Congo (DRC) (*1*–*4*). The illness manifests in multiple clinical forms, including bubonic and pneumonic plague. Pneumonic plague is rare, rapidly fatal, and transmissible from person to person via respiratory droplets. On March 4, 2019, a patient with suspected pneumonic plague was hospitalized in West Nile, Uganda, 4 days after caring for her sister, who had come to Uganda from DRC and died shortly thereafter, and 2 days after area officials received a message from a clinic in DRC warning of possible plague. The West Nile-based Uganda Virus Research Institute (UVRI) plague program, together with local health officials, commenced a multipronged response to suspected person-to-person transmission of pneumonic plague, including contact tracing, prophylaxis, and education. Plague was laboratory-confirmed, and no additional transmission occurred in Uganda. This event transpired in the context of heightened awareness of cross-border disease spread caused by ongoing Ebola virus disease transmission in DRC, approximately 400 km to the south. Building expertise in areas of plague endemicity can provide the rapid detection and effective response needed to mitigate epidemic spread and minimize mortality. Cross-border agreements can improve ability to respond effectively.

## Investigation and Findings

The index patient (patient A) was a Ugandan woman, aged 35 years, living in DRC, approximately 5 km from the Ugandan border. On February 27, 2019, Ugandan family members traveled to DRC for the funeral of patient A’s child, aged 4 years, and found patient A severely ill. They transported her to her ancestral Ugandan village in Zombo District of West Nile. While there, she complained of chest pain, experienced at least one episode of hemoptysis, and was admitted to a nearby clinic around midday the following day, February 28. She died a few hours later; no clinical samples were collected. She was buried in her ancestral village, preparation for which began the day of her death and culminated 2 days later, on March 2 (Table).

**TABLE:** Timeline of imported pneumonic plague transmission and public health response — Uganda, Feb 27–Mar 5, 2019

Date	Event
Feb 27	Ugandan family travels to the DRC for funeral and discovers patient A ill.
	Family transports patient A back to Uganda.
Feb 28	Patient A is cared for by patient B and others and transported to clinic in late morning.
	Patient A dies shortly after arrival.
Mar 1	Letter from DRC clinic arrives describing possible plague in the area where patient A resided.
Mar 2	Patient A is buried in her ancestral village in Uganda.
	UVRI plague team provides plague education to funeral attendees and begins area clinic plague refresher training.
Mar 3	Patient B experiences disease onset at approximately 11 a.m.
Mar 4	Patient B goes to clinic at approximately 9 a.m.; 8 hours later has difficulty breathing and coughs blood.
	Clinic staff members begin isolation measures, droplet precautions, and self-prophylaxis.
Mar 5	UVRI plague team and local officials perform additional contact tracing and administer prophylaxis to identified contacts.

**Abbreviations:** DRC = Democratic Republic of the Congo; UVRI = Uganda Virus Research Institute.

Meanwhile, on March 1, a local government office in Uganda received an alert from a private health clinic in DRC warning of possible plague circulation in a village near the border, the village from which patient A had come. Consequently, a team from UVRI’s plague program, along with local health officials, initiated plague education and risk communication at area health clinics and with village residents, in concert with the burial of patient A. Reportedly, her husband in DRC died of an acute illness at approximately the same time, and others in patient A’s family in DRC were ill, some with “fever and swellings.”

On March 3 in Uganda, patient B, aged 23 years (the sister of patient A), developed fever. In a health care facility the following day, she tested positive for malaria and lacked signs of pneumonia. She received intravenous artesunate for malaria, but in light of the suspicion for plague in the area, she was admitted and empirically started on gentamicin. Approximately 8 hours later, she coughed up blood-tinged sputum. Other patients were removed from the room, and droplet precautions were instituted.

Blood from patient B tested negative for Ebola virus disease and other hemorrhagic fever viruses at UVRI using established methods (*5*). Sputum yielded the maximal positive reaction (4+) on a commercial rapid diagnostic test (RDT) (New Horizons Diagnostics) for detection of *Yersinia pestis* fraction 1 (F1) antigen. Cultures of blood and sputum (obtained approximately 8 hours after initiation of antibiotic treatment) were negative. Subsequent testing of plasma and sputum by real-time polymerase chain reaction (PCR) yielded evidence of *Y. pestis* DNA. The patient was treated with gentamicin for 7 days and doxycycline for 4 days and was discharged on March 14. *Y. pestis* infection was confirmed by seroconversion on a total immunoglobulin F1 antigen passive hemagglutination assay (acute titer = 0 [collected March 4]; convalescent titer = 1:2,048 [collected March 18]).

Patient B did not travel to DRC for the burial of patient A’s child and did not arrive in the ancestral village to care for her sister until the morning of February 28. Patient B cared for patient A that morning, including using her hand to clean around patient A’s mouth, feeding her, transporting her to the clinic via motorbike, and attending to her at the clinic. She was not involved in transport of patient A’s body back to the village or in burial preparations.

## Public Health Response

On March 5, UVRI and district representatives rapidly mobilized and executed contact tracing and prophylaxis administration. In total, 129 persons were identified as contacts of patient A or B, including eight (6%) clinic staff members; 127 were placed on a 5-day prophylactic course of doxycycline, co-trimoxazole, or ciprofloxacin. Most persons identified as contacts (80; 62%) reported physical contact with or exposure within ≤1 m of either patient. Ninety-eight (76%) persons reported contact with patient A, including those involved in handling her body after her death. Fifty-three traced contacts (41%) had high-risk exposure as determined by subjective assessment of their distance from either patient and presumed patient infectiousness (Figure).

**FIGURE:**
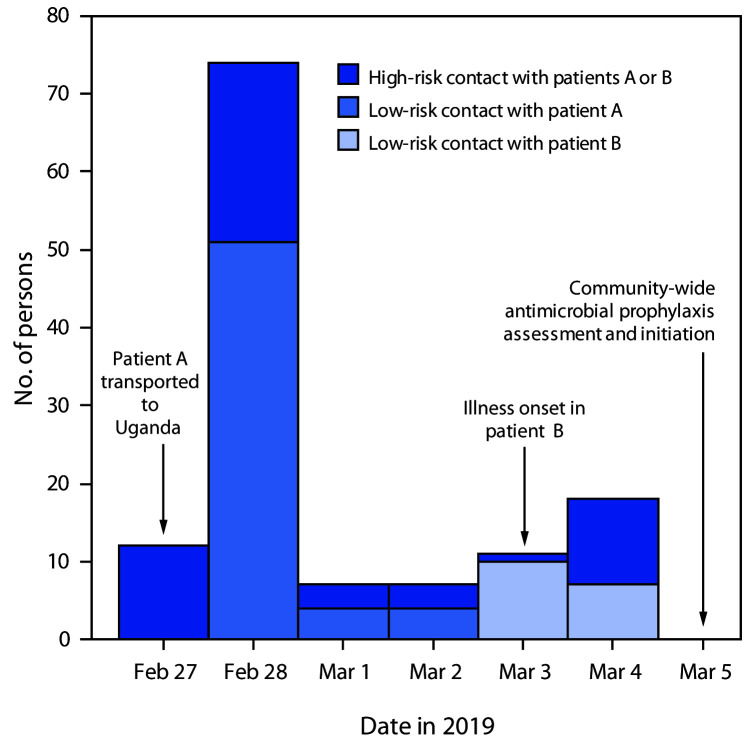
Number of persons exposed to patients A or B, by date, according to first reported exposure and assessment of pneumonic plague transmission risk — Uganda, 2019*^,†,§^ * High-risk contact with patients A or B includes transporting patient A via carrying or motorbike; caring for, washing, or feeding patient A on Feb 27 or Feb 28; physical manipulation of the body of patient A by washing, massaging, removing clothes, or dressing; providing health care or cleaning services related to patients A or B (until 48 hours after administration of antibiotics); coming in close and prolonged contact with patient B (e.g., sleeping in the same bed after illness onset or transporting to health facility). Figure reflects exposures among traced contacts; patient B is excluded from counts of persons with high-risk exposure to patient A. ^†^ Low-risk contact with patient A includes touching the body of patient A or briefly being in the same room as patient A. ^§^ Low-risk contact with patient B includes staying in the same room but at a distance during the day of illness onset, visiting her in the health care facility, or briefly touching her.

During a 10-day follow-up period, no identified contacts developed plague-like symptoms, and no indication of plague activity in Uganda was detected despite active clinic-, community-, and rodent-based surveillance for plague in the region. Comprehensive public health response was limited by jurisdiction; the UVRI team was unable to provide expertise and resources to support plague control just over the border in DRC. The fate of patient A’s DRC-based family and community members, given the likely ongoing circulation of *Y. pestis* among rodents and fleas in that village, is not known.

## Discussion

Plague persists in transmission cycles involving rodents and fleas on several continents, including Africa (*1*). Although plague generates fear because of its historical reputation, pneumonic plague transmission in modern times can be controlled by implementing droplet precautions, antimicrobial therapy, and prophylaxis of contacts (*6*,*7*). This report summarizes importation of plague from DRC into Uganda. Rapid and effective response curtailed epidemic spread of pneumonic plague beyond a single transmission event from patient A to patient B in Uganda.

Worldwide, most plague occurs following the bite of an infected flea and results in bubonic plague, characterized by acute fever and a painful swollen lymph node (*1*,*4*). Untreated, infection can spread to the lungs (*2*). Pneumonic plague transmission occurs via respiratory droplets and requires close contact with severely ill persons (*7*). The highest-risk exposures are those within 2 meters of persons coughing blood-tinged sputum; transmission might also occur during body preparation in traditional burials (*8*). The typical incubation period for primary pneumonic plague is <1 to 4 days, and the condition is often fatal if effective antibiotics are not initiated within 24–36 hours of illness onset (*2*).

Patient B’s exposure to patient A was limited to the morning hours of February 28 and was followed by patient B’s illness onset approximately 72 hours later. Persons with high-risk exposures to patient A as identified upon contact tracing were 3–5 days postexposure when antibiotic prophylaxis was initiated on March 5. Because only patient B became ill, the secondary attack rate among all persons with high-risk exposures was 2%. Postexposure prophylaxis might have prevented illness among some of those who received it, particularly those exposed to patient B, who were all still within the incubation period. This outcome highlights that pneumonic plague is not as transmissible as is often believed; and spread typically occurs among persons with close and substantial, rather than incidental, contact with a patient with late-stage disease (*7*). Secondary transmission rates in outbreaks in Madagascar and Uganda have been estimated at approximately 8%; however, transmission also depends on cultural and behavioral factors that might place persons at increased risk above the inherent transmissibility of the organism (*8*,*9*). Engagement with community leaders, members, health workers, and traditional healers in areas where plague is endemic can improve early recognition and implementation of simple interventions to curtail epidemic spread (*7*,*10*).

Even in areas with endemic plague, clinical diagnosis is challenging because of the nonspecific nature of the febrile illness in the absence of painful lymphadenopathy or blood-tinged sputum (*3*). RDT, real-time PCR, and paired serology testing were all positive for plague in patient B, despite collection of clinical specimens after initiation of effective antibiotic treatment, which did, however, hinder recovery of the organism in culture. RDT use occurred as part of ongoing research jointly conducted by CDC and UVRI to evaluate the sensitivity and specificity of RDTs for plague on human clinical specimens. Validated RDTs used by trained personnel might have value in providing rapid information to guide public health response but should be supported by additional diagnostic tests. Even in the remote setting of northwestern Uganda, collection of multiple clinical samples and use of multiple tests allowed for confirmation of the etiology.

CDC has worked with Uganda’s Ministry of Health and UVRI since 2003 to provide technical support for clinic- and animal-based plague surveillance, laboratory capacity, and community education and to conduct multifaceted research into improved diagnostics and effectiveness of environmental plague prevention approaches. Despite initial cross-border notification of suspected plague in DRC, lack of an established local cross-border collaboration prevented the resources and plague expertise in Uganda from supporting mitigation of ongoing risk just over the porous geopolitical boundary. Cross-border collaboration can improve capability to effectively respond to public health threats that affect border regions.

**Summary**

**What is already known about this topic?**

Plague is an acute zoonosis that occurs on several continents and can manifest in different clinical forms. Pneumonic plague is highly fatal and directly transmissible from person to person via infectious respiratory droplets.

**What is added by this report?**

Importation of pneumonic plague from the Democratic Republic of the Congo into an area of Uganda with effective public health response capabilities resulted in prompt action to halt transmission. Despite multiple high-risk exposures, only a single transmission event occurred.

**What are the implications for public health practice?**

Building expertise in areas of plague endemicity can provide the rapid detection and response needed to mitigate epidemic spread and minimize mortality. Cross-border agreements can improve ability to respond effectively.
